# Improving dietary citric acid production by the wild‐type *Aspergillus niger* ASP26 strain isolated from date by‐product

**DOI:** 10.1002/fsn3.4084

**Published:** 2024-04-08

**Authors:** Reda Bellaouchi, Ismail Hasnaoui, Meryem Idrissi Yahyaoui, Noureddine Bentouhami, Amina Hasnaoui, Mohamed Taibi, Amine Elbouzidi, Ahmad Mohammad Salamatullah, Hiba‐Allah Nafidi, Musaab Dauelbait, Mohammed Bourhia, Houssam Abouloifa, Yahya Rokni, Nabil Ghabbour, Ennouamane Saalaoui, Abdeslam Asehraou

**Affiliations:** ^1^ Laboratory of Bioresources, Biotechnology, Ethnopharmacology and Health, Faculty of Sciences Mohammed Premier University Oujda Morocco; ^2^ Laboratoire d'Amélioration Des Productions Agricoles, Biotechnologie et Environnement (LAPABE), Faculté Des Sciences Université Mohammed Premier Oujda Morocco; ^3^ Department of Food Science and Nutrition, College of Food and Agricultural Sciences King Saud University Riyadh Saudi Arabia; ^4^ Faculty of Agricultural and Food Sciences, Department of Food Science Laval University Quebec City Quebec Canada; ^5^ Faculty of Translation, Department of Scientific Translation University of Bahri Khartoum Sudan; ^6^ Laboratory of Biotechnology and Natural Resources Valorization, Faculty of Sciences Ibn Zohr University Agadir Morocco; ^7^ Research Unit of Microbiology, Biomolecules, and Biotechnology, Laboratory of Chemistry‐Physics and Biotechnology of Molecules and Materials, Faculty of Sciences and Techniques – Mohammedia Hassan II University of Casablanca Casablanca Morocco; ^8^ Research Unit Bioprocess and Biointerfaces, Laboratory of Industrial Engineering and Surface Engineering, National School of Applied Sciences, Sultan Moulay Slimane University Beni Mellal Morocco; ^9^ Laboratory of Natural Resources and Environment, Polydisciplinary Faculty of Taza Sidi Mohamed ben Abdellah University Taza Morocco

**Keywords:** *A. niger*, by‐product, citric acid, date, fermentation

## Abstract

This research investigates citric acid (CA) synthesis using the indigenous strain *Aspergillus niger* ASP26, which was isolated from date by‐products. The study initially involved isolating fungi capable of CA production and identifying the most potent strain based on its characteristic enzymatic activity. *A. niger* ASP26 was acknowledged in a previous study for its remarkable ability to produce extracellular enzymes, such as cellulase and amylase, which enable it to degrade organic materials effectively. After the identification phase, these isolates were screened for CA production using a modified Czapek‐Dox medium. The research identified significant factors affecting CA production in submerged fermentation, including pH, carbon source, inoculum size, and fermentation time. Optimal conditions were determined for *A. niger* ASP26, resulting in a maximum CA yield of 16.89 g/L. These conditions included a 2.5% spore suspension at 2 × 10^7^ spores/mL, an initial glucose concentration of 125 g/L, and incubation at 30°C for 144 h. Notably, *A. niger* ASP26 demonstrated the ability to produce CA under stress conditions as well. Citric acid is essential for various biological processes, such as cellular respiration, and is naturally present in citrus fruits. It also serves as a preservative and flavor enhancer in processed foods and beverages. The ability of *A. niger* ASP26 to produce CA from agricultural residues positions it as a viable candidate for sustainable CA production, harnessing the value from organic waste materials.

## Graphical abstract

Initially, fungi capable of producing citric acid were isolated and identified based on their characteristics and enzymatic activity. These isolates were then screened for CA production using a modified Czapek‐Dox medium. The study revealed significant effects of pH, carbon sources, inoculum size, and fermentation duration on CA production.

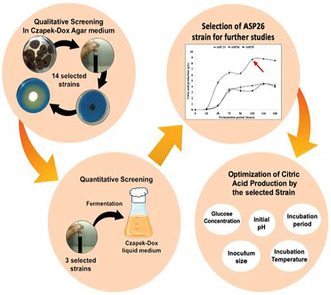

## 1 INTRODUCTION

Citric acid (CA) holds a crucial position in the chemical industry, with an annual production capacity reaching approximately 2 million tons (Karaffa & Kubicek, 2019). The sustained growth in demand for CA by the food, cosmetics, and pharmaceutical sectors, with an annual growth rate of 3–4% (Soccol et al., 2006), underscores its significance in various industrial applications.

CA is a naturally occurring, weak organic acid commonly found in many fruits and vegetables, with citrus fruits being particularly rich sources. It boasts a molecular weight of 210.14 g/mol and possesses three carboxylic functional groups, each with distinct pKa values (3.1, 4.7, and 6.4), profoundly influencing its solubility and stability across diverse environmental conditions. Within biological processes, CA serves as a primary metabolic product generated through the tricarboxylic acid cycle. Its production, predominantly achieved via fermentation, caters to the needs of industries such as food and beverage, pharmaceuticals and chemicals, textiles, and electroplating.

Notably, CA plays a multifaceted role in food product formulation, contributing as an acidulant, antioxidant, emulsifier, or preservative (Radwan et al., 2010; Soccol et al., 2006). Its versatile applications in these sectors stem from its advantageous attributes, encompassing low toxicity, high solubility, biodegradability, and pleasing taste characteristics (Ali et al., 2002). Despite its widespread use, CA's production predominantly relies on fermentation as the most cost‐effective and widely adopted method, representing over 90% of global CA production. However, this method falls short in meeting the surging demand for CA, necessitating the exploration of more cost‐efficient and sustainable production methodologies (Kim, 2004).

While numerous microorganisms, including fungi, bacteria, and yeasts, exhibit CA‐producing capabilities, only a select few can do so on an industrial scale (Soccol et al., 2006). Among these, *A. niger* stands out as the primary microbe chosen for large‐scale CA production due to its remarkable productivity, capability to ferment economical raw materials, ease of handling, and the ability to produce CA at low pH levels without releasing toxic metabolites (Lotfy et al., 2007). As a result, the primary objective of this study is to advance CA production through submerged cultivation techniques, harnessing *A. niger* strains sourced from date by‐products. This endeavor represents a pivotal step toward addressing the growing demand for CA while aligning with sustainability and cost‐effectiveness imperatives.

## 2 MATERIALS AND METHODS

### 2.1 Isolation and culturing of fungi strains

Thirty‐six *A. niger* (ASP) strains were isolated from date by‐products in a previous study (Bellaouchi et al., 2017). Each strain's spore solution was plated on Potato Dextrose Agar (PDA) medium (Biokar, France) and incubated at 25°C for 3–5 days. Subsequently, these strains were preserved on a slant PDA medium at 4°C and reactivated on PDA before utilization.

### 2.2 Inoculum preparation and standardization

The chosen *A. niger* strains, originally isolated from date by‐products, were reactivated on PDA medium and incubated at 25°C for 7 days. Following incubation, the spores were harvested using a solution of distilled water containing 0.1% Tween 80 (Sigma, St. Louis, MO, USA). The spore concentration was then adjusted to 2 × 10^7^ spores/mL using a hemocytometer (Thoma cell) for subsequent analysis.

### 2.3 Qualitative screening of CA production

#### 2.3.1 CA production using Czapek‐Dox agar medium

A qualitative screening was conducted to assess the CA production capability of 36 *A. niger* strains, following the procedure outlined by Ali (2004). This involved using the Czapek‐Dox Agar culture medium containing (g/L: Sucrose 30.0, NaNO_3_ 2.0, K_2_HPO_4_ 1.0, MgSO_4_.7H_2_O 0.5, KCl 0.5, FeSO_4_ 0.01, Bromocresol green 40 mL at a concentration of 1%, Agar 20, pH 6.0). 100 μL of the spore suspension of 2 × 10^7^ spores/mL was inoculated at the center of Petri dishes and incubated them at 25°C for 3–5 days. The development of a yellow halo around the colony was considered a positive result.

#### 2.3.2 Assessment of acidification capacity of *A. niger*

The acidification activity of 14 pre‐selected *A. niger* strains was assessed using the submerged fermentation method. We employed Czapek‐dox broth containing 50 g/L glucose, 2 g/L NaNO_3_, 1 g/L K_2_HPO_4_, 0.5 g/L MgSO_4_.7H_2_O, 0.5 g/L KCl, and 0.01 g/L FeSO_4_. This broth was inoculated with 1% (v/v) of a spore suspension at a concentration of 2 × 10^7^ spores/mL and adjusted to a pH of 5 using 4 M hydrochloric acid. The inoculated broth was then cultivated at 30°C under agitation at 200 rpm. The acidification activity of the selected strains was evaluated by measuring the pH at specific time points from 0 to 240 h of incubation.

### 2.4 Quantitative assessment of CA production

#### 2.4.1 Fermentation conditions

A submerged fermentation method was employed to quantitatively assess the ability of three selected *A. niger* strains (ASP 23, ASP 26, and ASP 29) to produce CA. A 1% (v/v) spore suspension of 2 × 10^7^ spores/mL was inoculated into Czapek‐dox broth, with pH adjustment to 5 using hydrochloric acid (4 M), followed by incubation at 30°C. pH, residual sugars, and CA levels were measured at 24‐h intervals over a 240‐h fermentation period, with an agitation speed of 200 rpm. The biomass growth of the strains was determined after the fermentation process.

#### 2.4.2 Measurement of pH

To evaluate the acidification activity of the three selected strains, the pH of the fermentation medium was measured every 24 h during the culture period using a calibrated VWR Symphony SB70P pH meter.

#### 2.4.3 Determination of residual sugars

Changes in residual sugars during fermentation were determined using the colorimetric DNS method. The optical density was measured at 523 nm using a spectrophotometer (UV‐1601). The intensity of the orange color generated was directly proportional to the content of reducing sugars (Miller, 1959). A calibration curve was prepared using standard glucose solutions ranging from 0 to 1.2 g/L.

#### 2.4.4 Measurement of CA concentration

CA concentration was determined using the colorimetric assay method developed by Marier and Boulet (1958). A 0.5 mL sample of the filtrate, obtained by centrifugation at 8000 *g* for 10 min, was mixed with 0.65 mL of pyridine and vigorously stirred before adding 2.85 mL of acetic anhydride. Tubes were incubated at 32°C for 30 min in a water bath. The optical density was measured at 420 nm, with the intensity of the color being directly proportional to the citrate concentration.

#### 2.4.5 Determination of biomass

The biomass of *A. niger* strains (ASP 23, ASP 26, and ASP 29) was determined by filtering the fermentation products through Whatman No. 1 filter paper and subsequently drying the wet biomass at 105°C until a constant weight was achieved.

### 2.5 Factors influencing CA production by *A. niger*

#### 2.5.1 Incubation period

The production of CA by *A. niger* strain ASP26 was monitored over a 336‐h incubation period. In a 250 mL Czapek‐dox broth adjusted to a pH of 5 using 4 M hydrochloric acid, we inoculated with 1% (v/v) of 2 × 10^7^ spores/mL of *A. niger* (ASP26) before incubating at 30°C. At the end of the incubation period, residual sugar, biomass growth, and CA concentration were determined.

#### 2.5.2 Initial pH

The impact of initial pH on CA production was assessed using 250 mL shake flasks containing 150 mL of Czapek‐dox broth, and adjusted to initial pH levels of 3, 4, 5, 6, and 7 using 4 M hydrochloric acid and 4 M NaOH, then inoculated with 1% (v/v) of 2 × 10^7^ spores/mL of *A. niger* (ASP26). A control culture was prepared with Czapek‐dox broth adjusted to pH = 5. Following inoculation, cultures were incubated at 30°C for 144 h, with CA concentration determined using the colorimetric assay method as previously mentioned.

#### 2.5.3 Incubation temperature

The effect of temperature on CA production was evaluated in 250 mL shake flasks containing 150 mL of liquid medium. *A. niger* (ASP26) spores were inoculated at a density of 2 × 10^7^ spores/mL (1%, v/v) into Czapek‐Dox broth. The cultures were then incubated at temperatures of 20°C, 25°C, 30°C, 35°C, and 40°C for a period of 144 h. Post‐incubation, the concentration of CA was quantified using the previously described colorimetric assay method.

#### 2.5.4 Glucose concentration

The effect of different glucose concentrations on CA production was evaluated in 250 mL shake flasks containing 150 mL of the Czapek‐dox broth containing 25, 50, 75, 100, 125, 150, 175, and 200 g/L of glucose was inoculated with 1% (v/v) of 2 × 10^7^ spores/mL of *A. niger* (ASP26). The CA concentration was determined using the above colorimetric assay after incubating the culture at 30°C for 144 h.

#### 2.5.5 Inoculum size

To assess the influence of inoculum size on CA production, *A. niger* (ASP26) was cultured in 250 mL shake flasks each containing 150 mL of Czapek‐Dox broth. The broth's pH was adjusted to 5.0 using 4 M HCl and 4 M NaOH. Inoculation was carried out with varying volumes of the spore suspension: 1%, 1.5%, 2%, 2.5%, and 3%, corresponding to a concentration of 2 × 10^7^ spores/mL. The cultures were incubated at 30°C for a duration of 144 h. Post‐incubation, CA concentration was quantified employing the earlier mentioned colorimetric assay method.

### 2.6 Optimization of CA production

The parameters for producing CA by *A. niger* (ASP26) in a modified Czapek‐dox broth were chosen based on previous optimal results. The broth contained 125 g/L glucose, 2 g/L NaNO_3_, 1 g/L K_2_HPO_4_, 0.5 g/L MgSO_4_.7H_2_O, 0.5 g/L KCl, and 0.01 g/L FeSO_4_, and was adjusted to an initial pH of 5 using 4 M hydrochloric acid and 4 M NaOH. The culture was inoculated with 2.5% of a spore suspension (2 × 10^7^ spores/mL) and incubated at 30°C for 192 h. The CA concentration was determined using a colorimetric assay method. A control experiment was also conducted using unmodified Czapek‐dox broth under the same conditions.

### 2.7 Statistical analysis

All experiments were conducted three times to ensure the validity of all the work carried out. The results were reported as means ± standard deviation. One‐way ANOVA analysis was employed to compare the means, with significance defined at *p* < .05. To identify groups of means, the Student–Newman–Keuls (S‐N‐K) post hoc test was utilized. All analyses were carried out using SPSS 10.0 for Windows (SPSS Inc., Chicago, USA).

## 3 RESULT AND DISCUSSION

### 3.1 Isolation of *A. niger* strains

In this study, a set of 36 strains of *A. niger* was successfully isolated from date by‐products and previously cataloged in Bellaouchi et al. (2021) prior to research. The meticulous selection of these particular strains played a pivotal role in shaping the research outcomes. This deliberate choice was underpinned by the strains' established capacity to produce enzymes crucial for decomposing organic matter, as highlighted in Bellaouchi et al. (2021) work. Specifically, these strains were handpicked due to their proficiency in generating amylase and cellulase enzymes, which are instrumental in breaking down complex carbohydrates, such as starch and cellulose. The scrupulous curation of these strains was imperative to ensure the optimal efficiency of the experimental procedures undertaken in our research.

### 3.2 Selection of *A. niger* producing CA

#### 3.2.1 Qualitative screening of CA production using Czapek‐Dox agar medium

**FIGURE 1:**
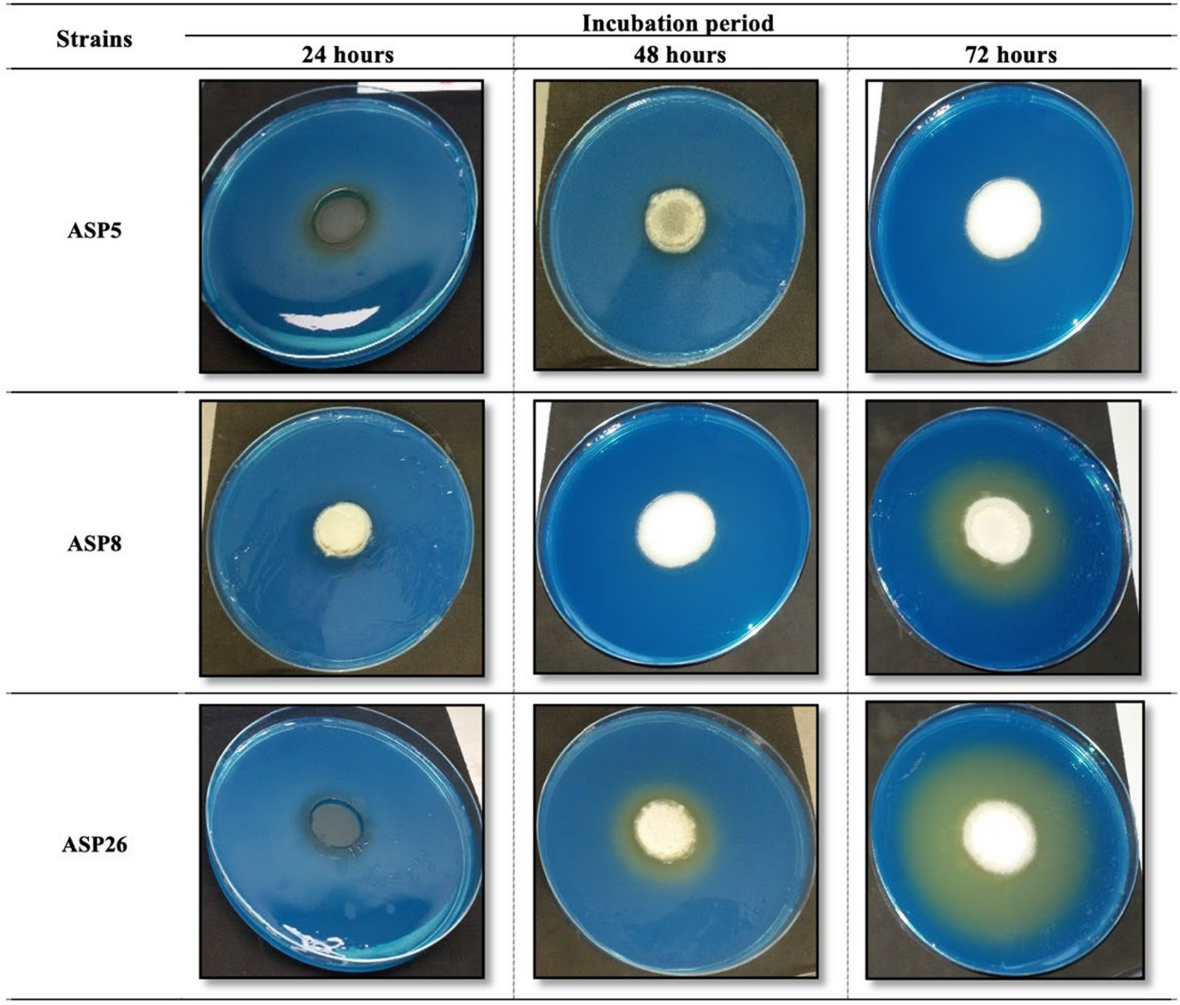
Qualitative screening of organic acids production, indicated by the yellow halo, by *Aspergillus niger* strains on Czapek‐Dox Agar medium.

**FIGURE 2:**
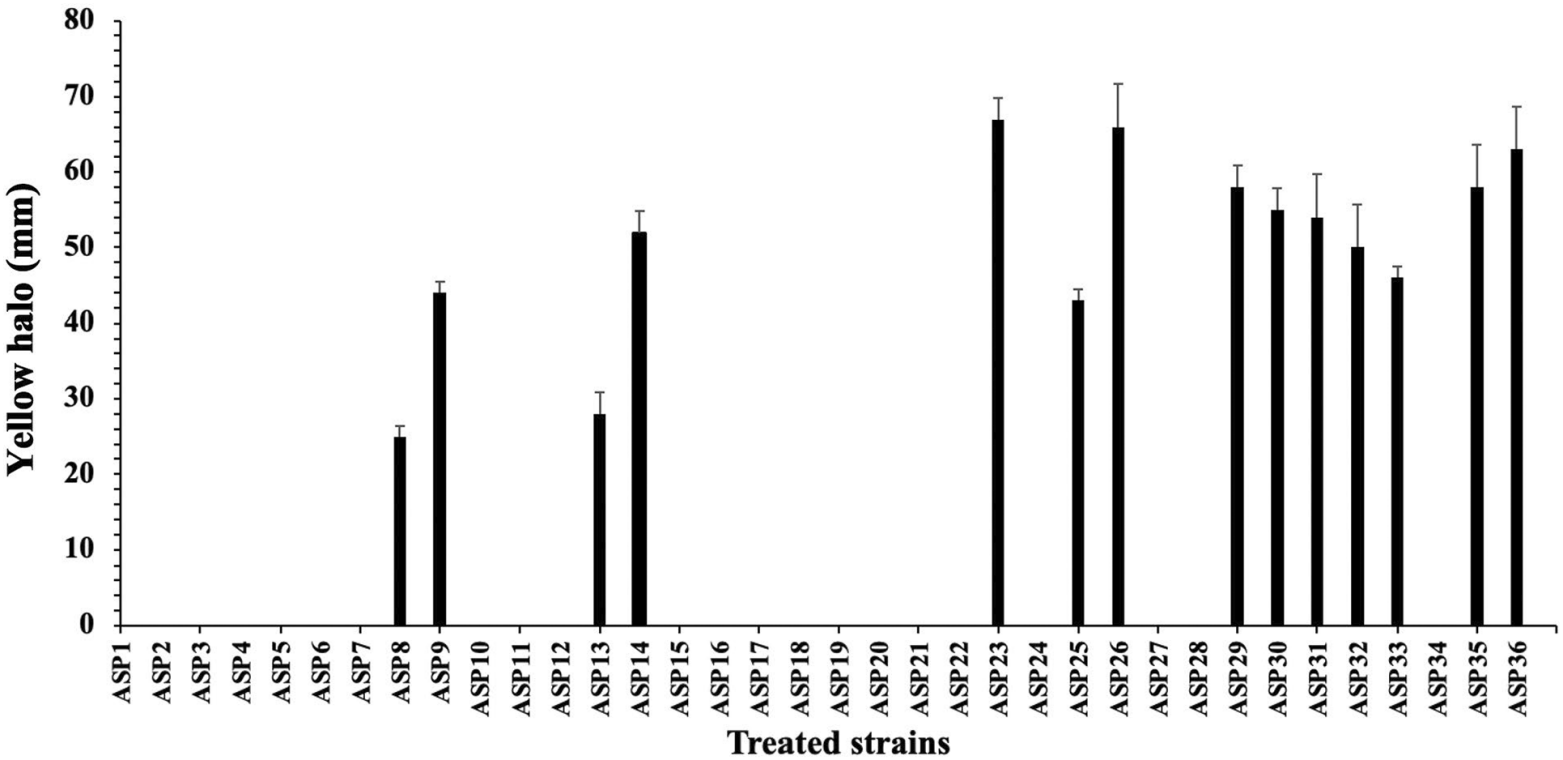
Diameter (mm) of yellow halo formation in strains treated on Czapek‐Dox Agar medium.

The qualitative screening of CA production by *A. niger* strains was based on their production of organic acids in the Czapek‐dox Agar medium supplemented with 0.04% Bromocresol purple (BCP). The color change from green‐blue to yellow, obtained for most of the *A. niger* strains, indicates their production capacity of organic acids. The yellow halo observed for some strains (ASP5, ASP8) is more intense than that obtained for others (ASP26) (Figure 1), indicating their higher production of organic acids. Among the 36 strains, 14 had a positive result (Figure 2), with a diameter of yellow halo ranging between 25 and 67 mm.

The results of the acidification activity of the selected *A. niger* strains obtained after 240 h of submerged fermentation using a Czapek‐Dox medium are presented in Table 1. The investigation disclosed that following a 24‐h incubation period, all strains manifested a substantial decrease in pH, ranging from an initial pH of 5 to a final range of 3.1–3.9. Furthermore, a significant decrease in pH (*p* < .001) was observed after 120 h of incubation for the three selected *A. niger* strains (ASP23, ASP26, and ASP29) with pH values of 1.9, 1.8, and 2.0, respectively (Table 1). This decrease in pH results from the acidification process caused by the breakdown of organic compounds in the Czapek‐Dox medium by the *A. niger* strains, which leads to the release of organic acids (Aboyeji et al., 2020). The high acidification potential of these strains makes them suitable for the quantitative analysis of CA production.

**TABLE 1:** pH changes during the culture of *Aspergillus niger* strains in Czapek‐Dox liquid medium at pHi 5, 200 rpm, and 30°C.

Strains	Incubation period (h)										
	0	24	48	72	96	120	144	168	192	216	240
ASP8	5.0	3.2 ± 0.00	2.6 ± 0.10	2.6 ± 0.00	2.8 ± 0.10	3.2 ± 0.20	2.6 ± 0.20	2.6 ± 0.20	2.6 ± 0.10	2.7 ± 0.00	2.7 ± 0.10
ASP9	5.0	3.5 ± 0.10	2.6 ± 0.10	2.5 ± 0.00	2.3 ± 0.10	2.2 ± 0.00	2.3 ± 0.00	2.3 ± 0.10	2.3 ± 0.00	2.4 ± 0.00	2.4 ± 0.10
ASP13	5.0	3.6 ± 0.10	2.6 ± 0.05	2.2 ± 0.10	2.1 ± 0.10	2.1 ± 0.00	2.2 ± 0.00	2.2 ± 0.10	2.3 ± 0.10	2.4 ± 0.00	2.3 ± 0.10
ASP14	5.0	3.5 ± 0.05	2.8 ± 0.10	2.7 ± 0.00	2.4 ± 0.10	2.3 ± 0.10	2.2 ± 0.00	2.1 ± 0.10	2.2 ± 0.10	2.1 ± 0.10	2.2 ± 0.00
ASP23	5.0	3.1 ± 0.00	2.2 ± 0.10	2.1 ± 0.10	2.1 ± 0.10	1.9 ± 0.10	2.2 ± 0.10	2.1 ± 0.00	2.2 ± 0.10	2.3 ± 0.10	2.3 ± 0.10
ASP25	5.0	3.9 ± 0.10	3.0 ± 0.20	2.5 ± 0.10	2.4 ± 0.00	2.3 ± 0.10	2.3 ± 0.00	2.4 ± 0.00	2.4 ± 0.10	2.4 ± 0.00	2.4 ± 0.10
ASP26	5.0	3.2 ± 0.10	2.2 ± 0.10	2.2 ± 0.00	2.2 ± 0.00	1.8 ± 0.10	2.1 ± 0.10	2.3 ± 0.10	2.3 ± 0.00	2.3 ± 0.10	2.3 ± 0.00
ASP29	5.0	3.3 ± 0.20	2.3 ± 0.30	2.0 ± 0.10	2.2 ± 0.00	2.0 ± 0.10	2.1 ± 0.10	2.2 ± 0.00	2.3 ± 0.10	2.3 ± 0.00	2.4 ± 0.20
ASP30	5.0	3.4 ± 0.20	2.5 ± 0.10	2.2 ± 0.10	2.1 ± 0.00	2.0 ± 0.10	2.2 ± 0.20	2.2 ± 0.10	2.3 ± 0.10	2.3 ± 0.00	2.3 ± 0.10
ASP31	5.0	3.6 ± 0.10	3.0 ± 0.10	2.6 ± 0.10	2.4 ± 0.10	2.2 ± 0.00	2.2 ± 0.10	2.2 ± 0.10	2.1 ± 0.00	2.2 ± 0.00	2.2 ± 0.10
ASP32	5.0	3.4 ± 0.10	2.8 ± 0.20	2.6 ± 0.30	2.2 ± 0.20	2.3 ± 0.10	2.3 ± 0.00	2.3 ± 0.10	2.4 ± 0.00	2.3 ± 0.20	2.4 ± 0.20
ASP33	5.0	3.4 ± 0.00	2.7 ± 0.20	2.6 ± 0.20	2.1 ± 0.10	2.1 ± 0.00	2.2 ± 0.00	2.2 ± 0.00	2.2 ± 0.10	2.3 ± 0.10	2.3 ± 0.00
ASP35	5.0	3.1 ± 0.10	2.5 ± 0.20	2.2 ± 0.10	2.1 ± 0.00	2.0 ± 0.10	2.1 ± 0.10	2.1 ± 0.10	2.2 ± 0.10	2.2 ± 0.20	2.3 ± 0.00
ASP36	5.0	3.3 ± 0.30	3.0 ± 0.10	2.7 ± 0.10	2.6 ± 0.00	2.4 ± 0.10	2.3 ± 0.00	2.2 ± 0.10	2.2 ± 0.00	2.2 ± 0.00	2.2 ± 0.10
Control (sterile culture medium)	5.0	5.0	5.0	5.0	5.0	5.0	5.0	5.0	5.0	5.0	5.0

#### 3.2.2 Quantitative screening and optimization of citric acid production by ASP26 strain in Czapek‐Dox broth

**FIGURE 3:**
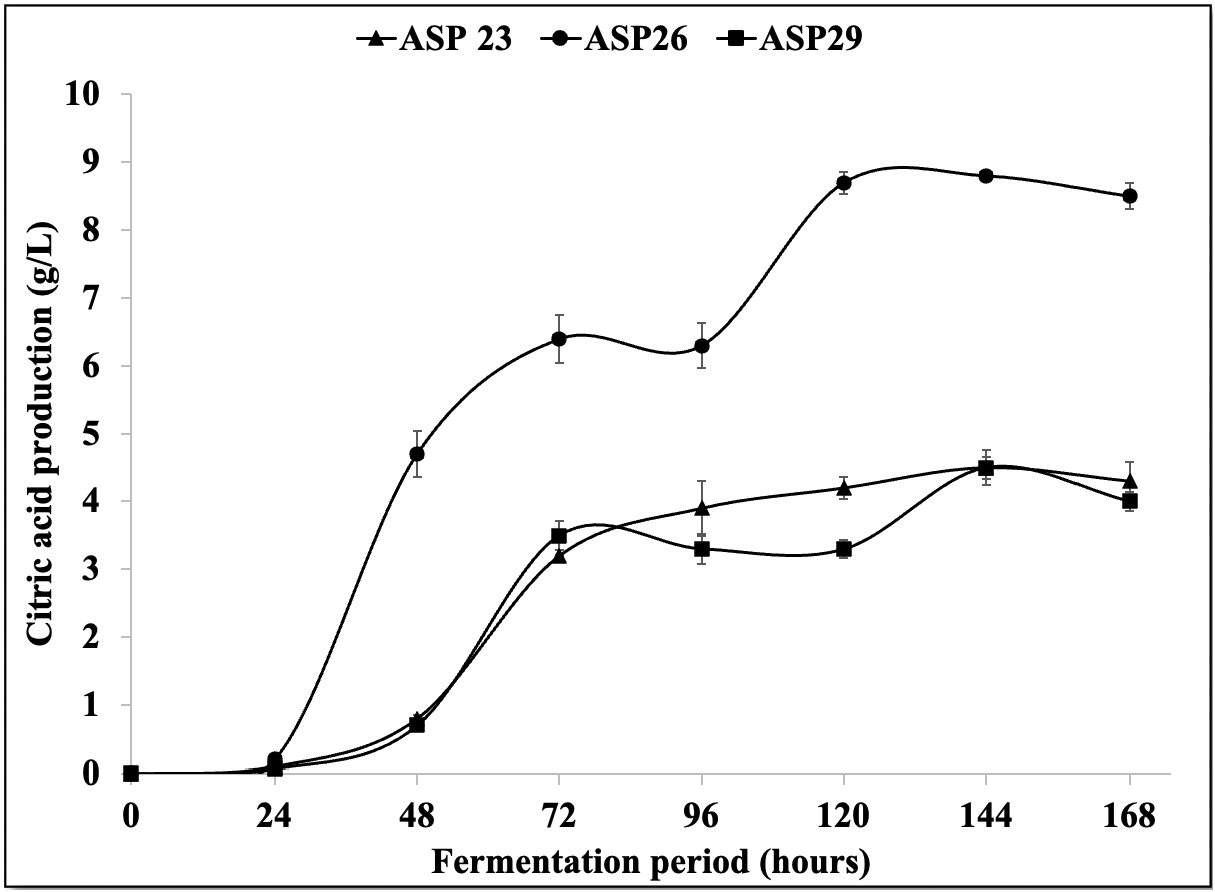
Citric acid production by *Aspergillus niger* strains (ASP23, ASP26, ASP29) in Czapek‐Dox liquid medium at pHi 5, 200 rpm, and 30°C.

**FIGURE 4:**
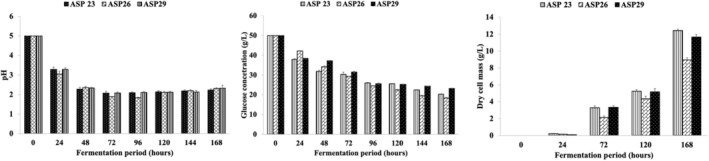
Evolution of pH, glucose concentration, and dry cell mass of *Aspergillus niger* strains (ASP23, ASP26, ASP29) during fermentation in liquid medium at pHi 5, 200 rpm, and 30°C.

The evaluation of CA production potential through submerged fermentation involved an examination of three strains of *A. niger*, namely, ASP23, ASP26, and ASP29. This comprehensive assessment encompassed the quantification of CA concentrations, pH dynamics, and residual sugar content, as graphically depicted in Figures 3 and 4. The outcomes revealed a noteworthy range in CA production among these strains, ranging from 4.5 to 8.8 g/L, accompanied by a mycelium dry weight varying between 8.9 and 12.4 g/L (Figure 4). Of particular significance was the ASP26 strain, which emerged as the most prolific CA producer, yielding an impressive 8.8 g/L. This represented a yield efficiency of 28.38% based on the consumed sugar. Furthermore, the fermentation process with ASP26 demonstrated a gradual decline in pH, reaching its nadir after 96 h. The mycelium dry weight reached its zenith at 8.9 g/L on the final day of fermentation, while residual sugars measured 18.37 g/L. In comparison, antecedent studies by Ali et al. (2001) delved into CA production by *A. niger* strains, revealing a diverse spectrum of CA concentrations ranging from 2.63 to 47.50 g/L and 0.24 to 16.04 g/L, respectively. The robust CA production observed with the ASP26 strain in our investigation aligns with and contributes to the expanding scholarly discourse on the diverse capabilities of *A. niger* strains in CA biosynthesis.

**FIGURE 5:**
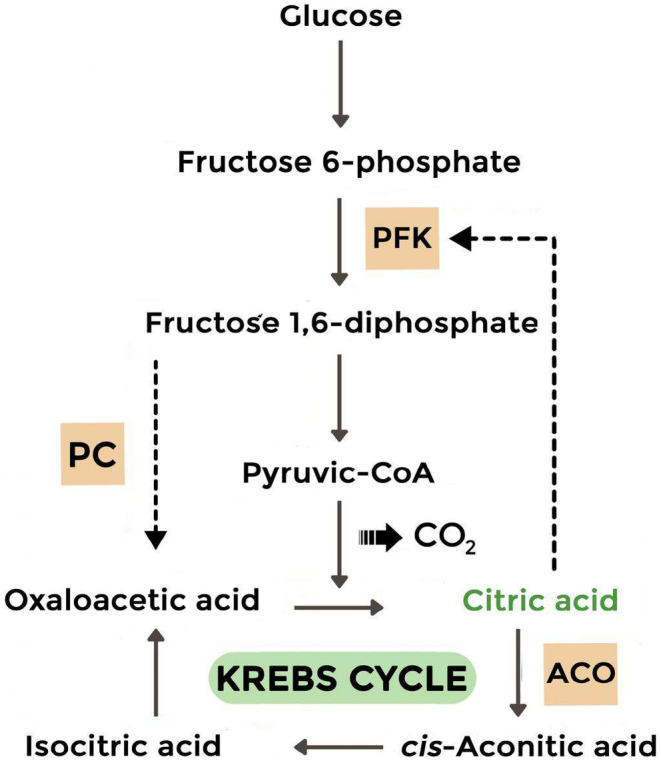
Schematic representation of the main metabolic reactions involved in citric acid production by *Aspergillus niger* (PC: phosphoénolpyruvate carboxykinase, ACO: Aconitase, PFK: phosphofructokinase).

The production of CA by *A. niger* is a complex process that depends on several factors, including the nutritional conditions of the growth medium. These conditions include the carbon source's concentration, dissolved oxygen level, hydrogen ions level, and optimal concentrations of phosphate and trace metals (Max et al., 2010; Soccol et al., 2006). The process of CA production is closely linked to the glycolytic pathway, and excessive production of citrate can occur under specific conditions. Research has shown that citrate can act as a glycolysis inhibitor; therefore, its inhibition has been a topic of interest. Under certain conditions, the inhibition of citrate can be reduced by the positive effects of the phosphofructokinase gene (PFK‐1) (Käppeli et al., 1978; Kristiansen & Sinclair, 1979). A manganese deficiency can also lead to protein breakdown, increasing the intracellular concentration of NH_4_^+^, known as the “ammonium pool.” This can inhibit the enzyme phosphofructokinase, essential for converting fructose and glucose to pyruvate, leading to an increased flux through glycolysis and CA formation (Figure 5). High glucose and NH_4_^+^ can also inhibit the formation of 2‐oxoglutarate dehydrogenase, ultimately limiting the catabolism of CA present in the tricarboxylic acid (TCA) cycle (Rohr & Kubicek, 1981).

### 3.3 Effect of initial pH and temperature on CA production

An experiment was undertaken to assess how initial pH values, ranging from 3 to 7, influenced CA production by the ASP26 strain in the Czapek‐Dox medium. The choice of this strain for further studies was based on its superior performance in CA production compared to other strains. The ASP26 strain showcased the highest CA yield, reaching 8.8 g/L, with an efficiency of 28.38% based on the consumed sugar. Moreover, the fermentation process demonstrated a gradual decline in pH, reaching its lowest point after 96 h. These significant findings establish ASP26 as the preferred strain for subsequent studies, underscoring its outstanding performance and potential for further exploration in our ongoing research. The results in Table 2 revealed very significant differences (*p* < .001) in CA production between the different pH values tested. It was reported that CA production by *A. niger* is inhibited under conditions leading to excessive biomass production (Baei et al., 2008). Thus, a maximum quantity of CA (8.22 g/L) was reached at an initial pH value of 5, with a lower value of the dry weight of the mycelium (4.34 g/L). The sugar consumed was 30.80 g/L, with a yield of 26.62%. When the initial pH value of the fermentation medium is greater than 5, the CA production gradually decreases, then drops sharply after the value of 6. However, Afifi (2011) indicated that the initial pH of 5.5 was optimal for CA production. Ali et al. (2002) reported a comparable pH value of 6 for maximum CA production using molasses as the fermentation substrate. El‐Hussein et al. (2009) reported a contradictory result and wrote a lower pH (3.5) for maximum CA production from molasses. Maintaining an optimal pH level is crucial for successful CA production. According to El‐Samragy et al. (1996), starting with a pH higher than 3.5 can cause the accumulation of oxalic and gluconic acids. Conversely, a pH below 2.5 can inhibit *A. niger* growth and ultimately affect CA production, as reported by Haq et al. (2002). The pH of the fermentation medium is a crucial factor in the biosynthesis of CA (Roukas, 2005). The pH of the medium plays a significant role during the two fermentation phases. A pH >5 is required for spore germination and mycelium growth in the initial phase. During the second phase, the CA production phase, a pH ≤2 is necessary for acid accumulation, to prevent contamination, and to inhibit the production of undesirable acids (oxalic and gluconic) (Papagianni, 2007). Roukas (2005) reported that when *Aspergillus niger* is used for CA production, the initial pH depends on the employed medium: in synthetic medium, the initial pH is adjusted to 2.5–3.5. Meanwhile, in a by‐product medium (molasses, fruit extract, syrup, etc.), the initial pH of the medium should be neutral or slightly acidic to ensure the germination and growth of the microorganism. Franz et al. (1993) found that *A. niger* produces CA at low pH in a synthetic medium, while *Penicillium simplicissimum* excretes CA at a higher pH range of 4–7. Furthermore, Papagianni (2007) noted that pH is crucial at two different stages of fermentation. Spores require a pH of 5 to germinate, and CA production requires a low pH of 2. Therefore, it is important to carefully determine the initial pH level to achieve maximum CA production (Soccol et al., 2006).

**TABLE 2:** Effect of initial pH (pHi) on citric acid production by *Aspergillus niger* ASP26 in submerged Czapek‐Dox liquid culture at 30°C and 200 rpm.

pHi	Citric acid, g/L	Residual sugar, g/L	Mycelium dry weight, g/L
3	5.11 ± 0.28^c^	16.14 ± 0.15^d^	5.14 ± 0.87^a^
4	4.32 ± 0.17^d^	14.80 ± 0.14^e^	4.89 ± 0.09^ab^
5	8.22 ± 0.13^a^	19.20 ± 0.56^c^	4.34 ± 0.18^b^
6	7.43 ± 0.17^b^	22.51 ± 0.26^b^	5.23 ± 0.15^a^
7	2.11 ± 0.04^e^	25.42 ± 0.45^a^	5.04 ± 0.32^a^

*Note*: Results are means ± SD (*n* = 3). Values of the same column, followed by the same letter, are not statistically different (*p* < .05) as measured by Student‐Newman‐Keuls test.

Very highly significant differences (*p* < .001) in CA production were observed at various temperatures (Table 3). Temperature plays a critical role in CA production, and our study found that the highest CA production (7.63 g/L) by the ASP26 strain occurred at an incubation temperature of 30°C. At this temperature, the mycelium's dry weight was 4.67 g/L, and the sugar consumption was 28.7 g/L, resulting in a CA yield of 27.52%. Other researchers have also reported that 30°C is optimal for CA production (Amer et al., 1999; El‐Hussein et al., 2009; Khosravi‐Darani & Zoghi, 2008).

**TABLE 3:** Influence of incubation temperature on citric acid biosynthesis by *Aspergillus niger* ASP26 in submerged Czapek‐Dox liquid culture at pHi 5 and 200 rpm.

T, °C	Citric acid, g/L	Citric acid yield, %	Sugar consumed, g/L	Mycelium dry weight, g/L
20	2.60 ± 0.24^c^	6.99 ± 0.19^e^	41.96 ± 0.27^a^	6.21 ± 0.13^a^
25	2.72 ± 0.37^c^	12.10 ± 0.19^c^	24.45 ± 0.18^d^	5.86 ± 0.12^b^
30	7.63 ± 0.44^a^	27.52 ± 0.24^a^	28.7 ± 0.32^b^	4.67 ± 0.25^d^
35	5.51 ± 0.37^b^	24.16 ± 0.40^b^	24.53 ± 0.28^d^	4.75 ± 0.26^d^
40	2.40 ± 0.24^c^	8.16 ± 0.05^d^	26.06 ± 0.29^c^	5.27 ± 0.22^c^

*Note*: Results are means ± SD (*n* = 3). Values of the same column, followed by the same letter, are not statistically different (*p* < .05) as measured by Student‐Newman‐Keuls test.

However, deviating from this temperature may reduce CA yield due to the denaturation of the citrate synthase enzyme and the activation of the oxalic acid synthesis pathway. Chioma and Agwa (2019) reported that the most favorable temperature for oxalic acid production was 35°C, but it completely inhibited the accumulation of CA at this temperature. Therefore, maintaining an incubation temperature of 30°C is crucial for achieving optimal CA production.

### 3.4 Effect of the incubation period on the CA production

**FIGURE 6:**
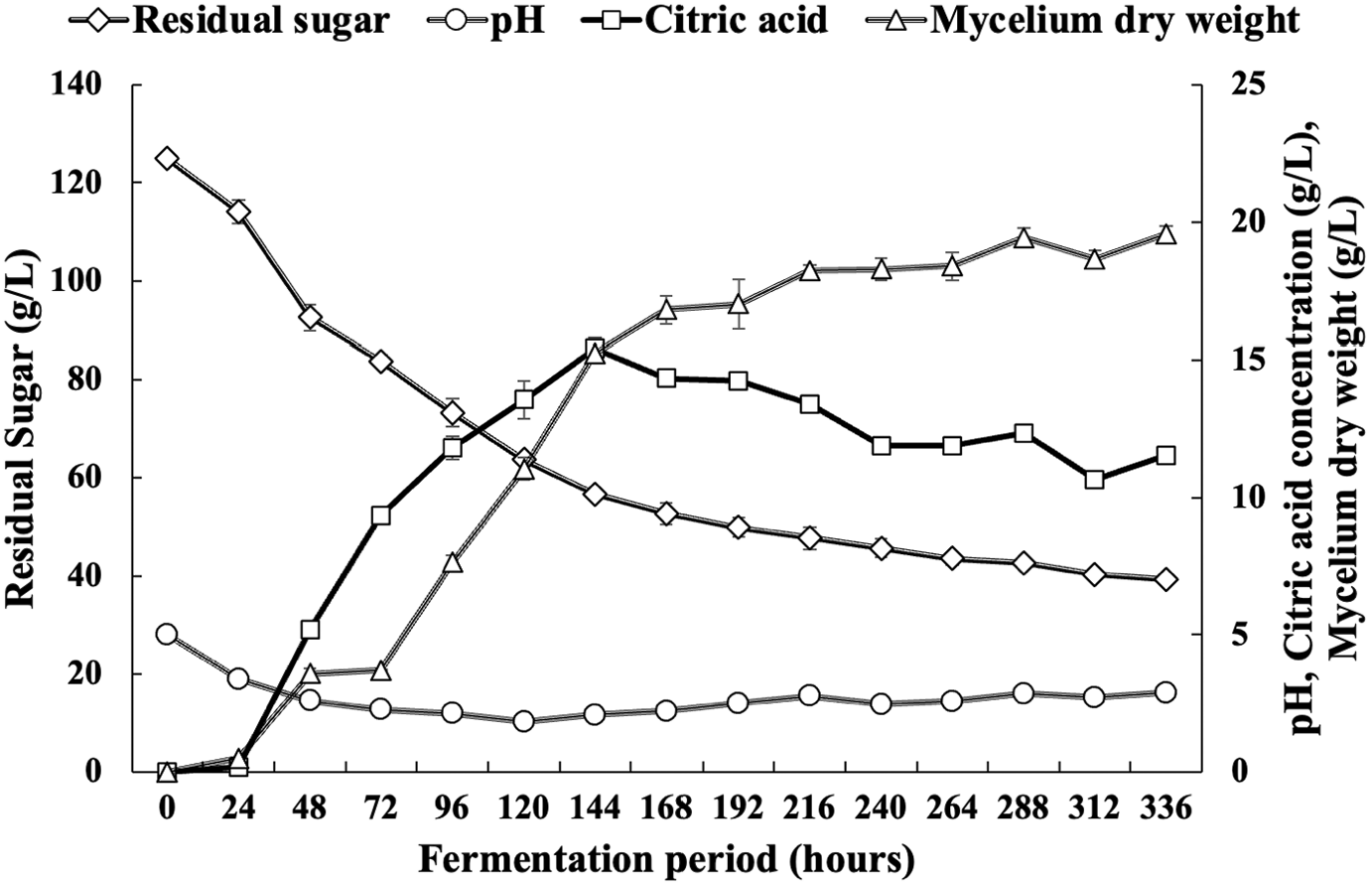
Biochemical (pH, citric acid, sugars) and biomass (mycelium dry weight) changes during the submerged culture of *Aspergillus niger* ASP26 strain.

To investigate the effects of the incubation period on CA production, a fermentation process was carried out using the selected strain ASP26 for 336 h, and the results are presented in Figure 6. Highly significant differences (*p* < .001) in CA production were observed during the incubation period (Figure 6). The study noted that the biosynthesis rate of CA production increases gradually during fermentation, reaching its peak value of 15.43 g/L at 144 h post‐inoculation. These findings are consistent with previous studies by Ali et al. (2002) who also stated that the highest productivity of CA is attained following 144 h of fermentation. In contrast, researchers like Arzumanov et al. (2000), Alvarez et al. (2007), Lotfy et al. (2007), and Nadeem et al. (2010) reported that the maximum concentration of CA was obtained after 192 h of fermentation. At the optimal CA production with ASP26 strain, the corresponding pH, residual sugar, and mycelial dry weight values were 2.08, 56.66, and 15.25 g/L, respectively (Figure 6). Increasing the fermentation time did not increase the production of an additional amount of CA. Consequently, a decrease in productivity may be due to the reduction of the nitrogen available in the fermentation medium, the age of the strain, the exhaustion of sugar contents, and the degradation of the enzymatic system responsible for CA biosynthesis.

### 3.5 Effect of glucose concentration on CA production

The isolated *A. niger* ASP26 was cultivated on the Czapek‐Dox medium with glucose concentrations ranging between 25 and 200 g/L. Highly significant differences (*p* < .001) in CA production were observed between glucose concentration values (Table 4). The maximum concentration of CA (15.46 g/L) was obtained with an initial glucose concentration of 125 g/L. The dry weight of the mycelium at this concentration was 12.13 g/L, while the sugar consumed was 59.69 g/L, with a yield of 25.79%. An increase or decrease in the initial glucose concentration above 125 g/L would decrease the yield of CA produced. This can be explained by the formation of oxalic acid and polyalcohols (Haq et al., 2003). The presence of elevated concentrations of carbon sources frequently leads to the inhibition of α‐ketoglutarate dehydrogenase (α‐KGDH) activity (Papagianni, 2007). This enzyme plays a crucial role in the CA (Krebs) cycle by facilitating the conversion of α‐ketoglutarate into succinyl‐CoA. With ample carbon sources, the cell often prioritizes other metabolic pathways, such as glycolysis, at the expense of the CA cycle. Consequently, α‐KGDH activity tends to be downregulated when exposed to high carbon source concentrations (Papagianni, 2007). It is hypothesized that citric acid production (CA) by *A. niger* during fermentation can be influenced by the physiological stress experienced by the cells, particularly in response to the specific carbon source utilized. This stress‐driven response may divert cellular resources toward CA production, potentially at the cost of mycelial growth (biomass). Notably, as the glucose concentration exceeds 125 g/L, the strain seems to prioritize biomass formation over CA production, gradually decreasing CA concentration. This observation underscores the significance of the carbon source's type and concentration in modulating the cellular response. Thus, it underscores the critical importance of judicious carbon source selection when enhancing the efficiency of CA production.

**TABLE 4:** Effect of initial glucose concentration on citric acid biosynthesis by *Aspergillus niger* ASP26 in submerged Czapek‐Dox liquid culture at pHi 5, 200 rpm, and 30°C.

Glucose, g/L	Citric acid, g/L	Residual sugar, g/L	Mycelium dry weight, g/L
25	0.22 ± 0.04^h^	11.27 ± 0.37^h^	6.13 ± 0.08^f^
50	8.45 ± 0.32^e^	22.24 ± 0.48^g^	5.21 ± 0.33^f^
75	8.57 ± 0.24^e^	35.41 ± 0.72^f^	11.11 ± 0.08^e^
100	10.23 ± 0.09^c^	49.09 ± 0.62^e^	14.67 ± 0.17^c^
125	15.46 ± 0.16^a^	65.31 ± 0.50^d^	12.13 ± 0.41^de^
150	12.04 ± 0.23^b^	74.69 ± 1.15^c^	12.64 ± 0.87^d^
175	7.87 ± 0.50^f^	96.07 ± 0.52^b^	18.49 ± 1.58^b^
200	4.43 ± 0.76^g^	97.21 ± 0.95^a^	25.64 ± 1.55^a^

*Note*: Results are means ± SD (*n* = 3). Values of the same column, followed by the same letter, are not statistically different (*p* < .05) as measured by Student‐Newman‐Keuls test.

### 3.6 Effect of inoculum size on CA production

The *A. niger* ASP26 strain was inoculated on a Czapek‐Dox medium with different inoculum volumes ranging from 1% to 3%. Table 5 indicates the impact of the inoculum volume on the production of CA, the sugar consumed, and the dry weight of the mycelium. Highly significant differences (*p* < .001) in CA production were observed between the inoculum size values. A maximum of CA (16.89 g/L) was obtained when 150 mL of the culture medium were inoculated with 3.75 mL (2.5%) of spore suspension with a 2 × 10^7^ spores/mL concentration. Ali et al. (2002) obtained the maximum CA from molasses using an inoculum rate of 1%, while Bari et al. (2009) obtained a maximum concentration of CA from 10% palm fruit clusters. High inoculum density can result in overcrowding, heightened competition, and the rapid depletion of nutrients (Uyar & Baysal, 2004). Typically, the production of metabolites tends to increase with inoculum density up to a certain threshold (Nampoothiri et al., 2004). Conversely, at low inoculum density, metabolite production declines, and the risk of contamination rises due to an inadequate cell population (Adham, 2002; Ruijter et al., 2000).

**TABLE 5:** Effect of inoculum size on citric acid biosynthesis by *Aspergillus niger* ASP26 in submerged Czapek‐Dox liquid culture at pHi 5, 200 rpm, initial glucose concentration of 125 g/L, incubated at 30°C.

Inoculum, %	Citric acid, g/L	Residual sugar, g/L	Mycelium dry weight, g/L
1	14.14 ± 0.59^c^	69.63 ± 1.16^d^	15.21 ± 1.07^c^
1.5	14.48 ± 1.08^bc^	75.34 ± 1.15^a^	17.62 ± 0.08^b^
2	15.23 ± 0.20^b^	73.24 ± 1.14^c^	16.86 ± 0.09^b^
2.5	16.89 ± 0.23^a^	68.57 ± 1.19^d^	17.42 ± 0.21^b^
3	14.44 ± 0.43^bc^	74.40 ± 0.55^bc^	19.64 ± 0.10^a^

*Note*: Results are means ± SD (*n* = 3). Values of the same column, followed by the same letter, are not statistically different (*p* < .05) as measured by Student‐Newman‐Keuls test.

## 4 CONCLUSION

The present study highlights the critical role of key factors, including initial sugar concentration, pH levels, and specific fermentation conditions, in citrate production. Among the 36 strains of *A. niger* studied, the wild‐type ASP26 isolate demonstrated remarkable performance in producing CA within the fermentation medium. Under controlled conditions, maintaining an initial pH of 5 and utilizing the ASP26 strain, we achieved a citrate concentration of 8.22 g/L. This achievement soared to an impressive 16.89 g/L when inoculating the culture medium with a 2.5% spore suspension at 2 × 10^7^ spores/mL. These conditions involved an initial glucose concentration of 125 g/L, a temperature of 30°C, and a 144‐h fermentation period. Despite these exciting results, there is ample room for improvement. Future research should explore alternative high‐efficiency strains and fine‐tune additional factors influencing the fermentation process in *A. niger* to boost citrate production further. Notably, the present findings indicate that CA overproduction is linked to stress conditions experienced by the strain. This discovery opens up exciting possibilities, as these stress‐induced mechanisms could be harnessed to enhance metabolite production not only in *A. niger* but potentially in other microorganisms as well. Stress factors, including substrate requirements, biomass management, and product yield, deserve careful attention as we strive to optimize the fermentation process and pioneer new frontiers in bioprocessing.

## AUTHOR CONTRIBUTIONS

**Reda Bellaouchi:** Writing – review and editing; **Ismail Hasnaoui:** Resources; **Meryem Idrissi Yahyaoui**: Methodology; **Noureddine Bentouhami:** Software; **Amina Hasnaoui:** Supervision; **Mohamed Taibi:** Data curation; **Amine Elbouzidi:** Formal Analysis; **Ahmad Mohammad Salamatullah:** Investigation, Funding acquisition; **Hiba‐Allah Nafidi:** Visualization, Funding acquisition; **Musaab Dauelbait:** Writing – original draft; **Mohammed Bourhia:** Data curation; **Houssam Abouloifa:** Formal Analysis, **Yahya Rokni:** Investigation; **Nabil Ghabbour:** Visualization; **Ennouamane Saalaoui:** Project administration; **Abdeslam Asehraou:** Validation.

## FUNDING INFORMATION

This work is also financially supported by the Researchers Supporting Project number (RSP‐2024R437), King Saud University, Riyadh, Saudi Arabia.

## CONFLICT OF INTEREST STATEMENT

The authors disclose that there are no conflicts of interest.

## ETHICS STATEMENT

This research does not entail experimentation on humans or animals.

## Data Availability

Data are available upon request from the corresponding authors.
